# Tuberculosis of the Parotid Gland

**DOI:** 10.1155/2012/278793

**Published:** 2012-08-08

**Authors:** Vivek Gupta, Kiran Patankar, Archana Shinde, Charu Bhosale, Ajitey Tamhane

**Affiliations:** Department of Radiology, Apple Hospital, Kolhapur 416001, India

## Abstract

Parotid gland involvement is extremely rare, even in countries in which tuberculosis is endemic. Clinically, it usually presents as a slow-growing mass indistinguishable from a malignancy. On imaging too, tuberculosis of the parotid may mimic neoplasm. The diagnosis of parotid tuberculosis needs a high degree of clinical suspicion. This paper highlights the clinical presentation, imaging findings, and importance of FNAC in diagnosis of this rare entity.

## 1. Case Presentation

A 15-year-old male presented with a gradually enlarging swelling in the left parotid region of one year duration. It was associated with mild pain in this region. He had caries of the upper molar teeth on the ipsilateral side. There was no history of fever or night sweats on presentation. His past history was unremarkable. He gave no history of personal or family history of tuberculosis. 

Physical examination revealed a mobile lobulated swelling in the region of the left parotid gland. There were no overlying secondary skin changes. There were no visible fistulae or sinuses. Facial nerve function was intact. On palpation, a firm, mobile and nonfluctuant 3 cm area of mass-like enlargement was noted involving the left parotid gland. There was palpable cervical lymphadenopathy. The right parotid area was clinically normal. On investigation, the hemogram including erythrocyte sedimentation rate and chest radiograph were normal.

**Figure 1:**
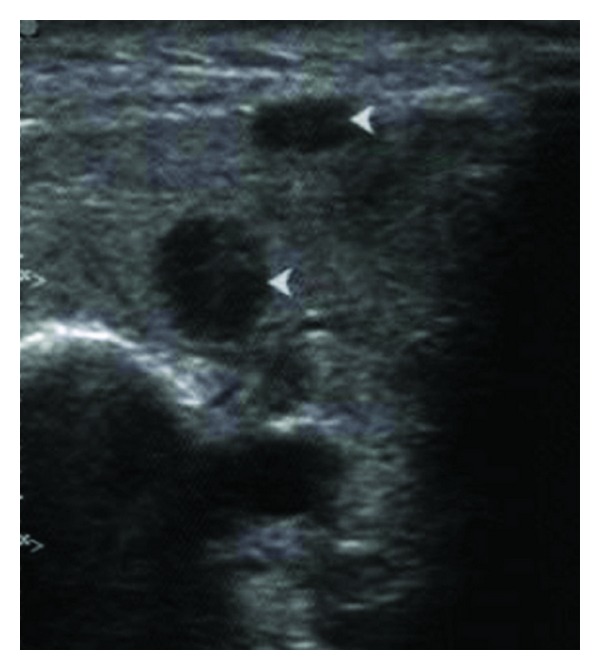
The left parotid. Small well-defined hypoechoic lesions (arrowheads) are seen within the parenchyma of the relatively hyperechoic left parotid gland.

**Figure 2:**
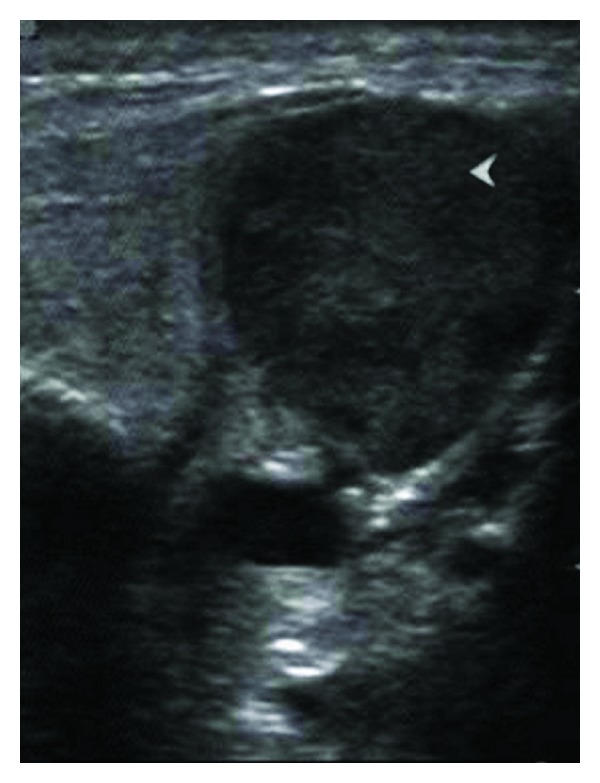
The left parotid. A large well-defined hypoechoic lesion (arrowhead) within the left parotid.

**Figure 3:**
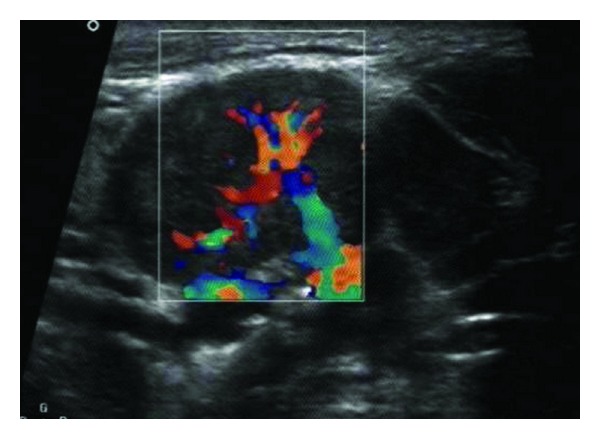
Lesion within left parotid on colour doppler study; shows significant vascularity.

**Figure 4:**
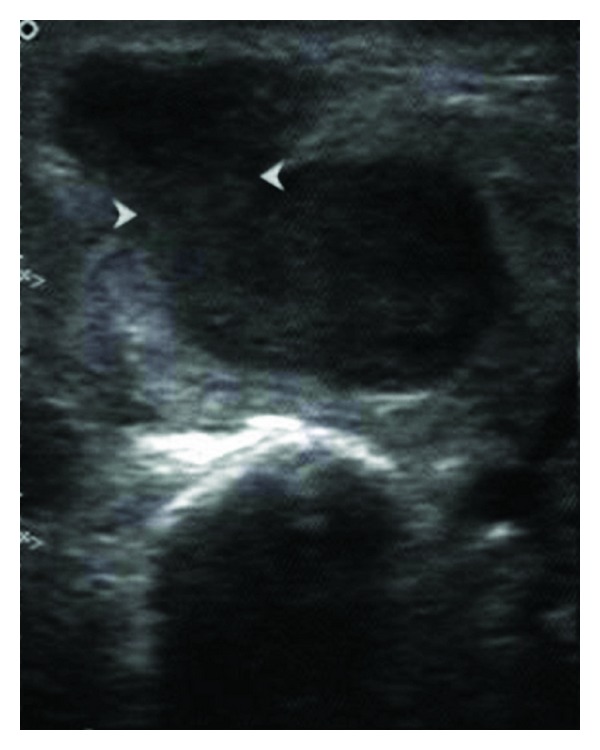
One of the hypoechoic lesions is protruding to the exterior of the gland parenchyma. The arrowheads show the surface breach through which the lesion has entered the extraglandular space.

**Figure 5:**
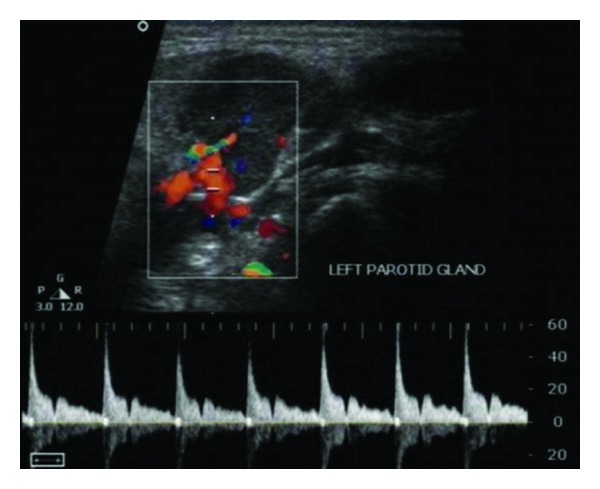
Colour doppler spectral waveform within the intralesional flow shows arterial type of flow with relatively high resistance.

An ultrasound (US) of the left parotid area was advised. On US, the left parotid gland appeared enlarged in size. Few well-defined, hypoechoic oval mass lesions were noted within the substance of the left parotid gland. These lesions were quite obvious against the relatively hyperechoic appearance of the normal parotid parenchyma (Figure 1). The largest lesion measured approximately 1.8 × 1.2 cm (Figure 2). There were no calcifications or ductal dilatation. One of the intraparenchymal lesions quite close to the gland surface was noted bulging into the extraglandular space through a breach in the gland surface (Figure 4). On colour doppler study, there was significant vascularity noted within the lesions (Figure 3). This vascularity showed a relatively high resistance type of arterial spectral waveform with PSV reaching upto 60 cm/s (Figure 5). Enlarged lymph nodes were noted in the left submandibular region (level Ib) and in the upper cervical region (level II). These lymph nodes showed significant vascularity within them on colour doppler study. The largest lymph node measured 2.8 cm in short axis. Though preserved fat hilum could be identified in the relatively smaller involved lymph nodes in this region, the largest lymph nodes appeared devoid of fat hilum. A provisional diagnosis of either infection or neoplasm was given. Since the lesion was quite accessible, US guided FNAC was advised.

Fine needle aspiration cytology was performed under US guidance. Cytology showed granulomas made up of epithelioid cells and occasional giant cells surrounding caseous type of necrosis. Scattered abscesses were also seen. No malignant cells were detected. The FNAC opinion was chronic caseating granulomatous inflammation of salivary gland consistent with tuberculosis. Tuberculin test was done which turned out to be strongly positive. The patient was advised prolonged antitubercular medication. 

## 2. Discussion

Tuberculosis is a necrotizing granulomatous disease with varied clinical presentations and a wide distribution. The lungs are most commonly involved. Extra thoracic forms of the disease account for approximately 20% of overall active tuberculosis and can be seen in the kidneys, bones, meninges, and lymph nodes [1]. Although tuberculosis is common in countries like India, tuberculous infection of parotid gland is uncommon [2].

Involvement of the parotid gland and lymph nodes may develop in two ways. First, a focus of mycobacterial infection in the oral cavity liberates the mycobacterium that ascends into the salivary gland via its duct or pass to its associated lymph nodes via lymphatic drainage. The second pathway involves haematogenous or lymphatic spread from a distant primary lung focus [1]. Tuberculous involvement of the salivary glands is more commonly seen secondary to systemic dissemination of pulmonary tuberculosis than as primary extrapulmonary tuberculosis. If the salivary glands are primarily affected, the parotid gland is involved 70% of the time [3]. 

Tuberculosis of the parotid gland presents with difficulties in diagnosis because of the similarity of the presentation to that of a neoplasm. Diagnosis mainly relies on the treating physician having a high index of suspicion. 

Kant and associates in 1977 reported 35 cases [4]; Yaniv and Avedillo added 2 more cases in 1985 [5] and Sharma et al. reported yet another case in 1996 [6]. Only about a hundred cases have been reported till 2003 and mostly on parotidectomy specimens [7]. 

Radiological investigations including US, CT, and MRI are sensitive in detecting intraparotid tubercular lesions, however, findings are not specific. Most of the imaging findings mimic neoplasm. A chest radiograph may be helpful in cases of associated pulmonary tuberculosis. The chest radiograph of our patient was normal. US may well delineate the lesion however the echotexture has been variably described in literature most commonly being hypoechoic as in our case. The colour doppler findings within the lesions are also not specific and may range from avascular to highly vascular lesions. CT and MRI are useful to define the extent of the lesion and detect any concomitant deeper lesions. Again the findings on CT and MRI are highly sensitive however not specific and may be indistinguishable from neoplasm. In patients presenting with unilateral parotid nodules, tuberculosis should be considered when linearly arranged enhancing nodules are demonstrated in the superficial lobes of the glands on CT scan [8]. On MRI, the lesions usually appear hypointense on T1 and hyperintense on T2 weighted images with homogenous contrast enhancement which is a nonspecific finding [9]. Since the imaging findings are indistinguishable from neoplasm, most cases may undergo surgery like superficial parotidectomy and diagnosis being established after surgery. 

Establishment of diagnosis of tuberculosis requires histological confirmation since imaging findings are not specific. Tuberculous disease is impossible to distinguish clinically from other diffuse inflammatory disease of the salivary glands if a culture of the glandular secretions from the Stenson's duct or saliva are negative for AFB [10]. In parotid lesions FNAC has a sensitivity of 81–100% and specificity of 94–100% [11]. Thus, FNAC should be performed first in the evaluation of a parotid mass [9]. However FNAC findings may turn out to be inconclusive or inadequate and such cases may undergo inadvertent surgery.

In our patient, the most probable source of infection for the parotid gland was via the Stenson's duct from the oral cavity since he had molar caries in the ipsilateral side and no other tubercular focus could be established elsewhere in the body. The diagnosis of tuberculosis of the parotid was clinched due to FNAC of the intraparotid lesions under US guidance. CT or MRI was not indicated in this case. Our case is unique as most previously described case reports of tuberculosis of the parotid have been achieved post surgery on parotidectomy specimens where initial FNAC had failed to achieve diagnosis. In our case since the FNAC was conclusive, surgery was avoided, and management was conservative. This highlights the importance of ancillary investigations like FNAC in such cases. Although rare, tuberculosis should be kept in mind and considered in the differential diagnosis of any patient presenting with a solitary swelling in the parotid gland to avoid unnecessary surgery [9].
